# USP47-Mediated Deubiquitination and Stabilization of TCEA3 Attenuates Pyroptosis and Apoptosis of Colorectal Cancer Cells Induced by Chemotherapeutic Doxorubicin

**DOI:** 10.3389/fphar.2021.713322

**Published:** 2021-09-23

**Authors:** Xiaodan Hou, Jun Xia, Yuan Feng, Long Cui, Yili Yang, Peng Yang, Xin Xu

**Affiliations:** ^1^ Suzhou Institute of Systems Medicine, Center for Systems Medicine, Chinese Academy of Medical Sciences, Suzhou, China; ^2^ Department of Emergency Medicine, the First Affiliated Hospital of Soochow University, Suzhou, China; ^3^ Department of Colorectal and Anal Surgery, Xinhua Hospital, Shanghai Jiao Tong University School of Medicine, Shanghai, China; ^4^ China Regional Research Centre, International Centre of Genetic Engineering and Biotechnology, Taizhou, China

**Keywords:** USP47, TCEA3, pyroptosis, apoptosis, colorectal cancer

## Abstract

The ubiquitin–proteasome system regulates a variety of cellular processes including growth, differentiation and apoptosis. While E1, E2, and E3 are responsible for the conjugation of ubiquitin to substrates, deubiquitinating enzymes (DUBs) reverse the process to remove ubiquitin and edit ubiquitin chains, which have profound effects on substrates’ degradation, localization, and activities. In the present study, we found that the deubiquitinating enzyme USP47 was markedly decreased in primary colorectal cancers (CRC). Its reduced expression was associated with shorter disease-free survival of CRC patients. In cultured CRC cells, knockdown of USP47 increased pyroptosis and apoptosis induced by chemotherapeutic doxorubicin. We found that USP47 was able to bind with transcription elongation factor a3 (TCEA3) and regulated its deubiquitination and intracellular level. While ectopic expression of USP47 increased cellular TCEA3 and resistance to doxorubicin, the effect was markedly attenuated by TCEA3 knockdown. Further analysis showed that the level of pro-apoptotic Bax was regulated by TCEA3. These results indicated that the USP47-TCEA3 axis modulates cell pyroptosis and apoptosis and may serve as a target for therapeutic intervention in CRC.

## Introduction

Ubiquitination regulates the level, localization, and activity of target proteins and affects diverse cellular processes, including cell growth, differentiation and apoptosis (Melino 2005; Bernassola et al., 2008). The modification usually requires the sequential actions of ubiquitin-activating enzyme (E1), ubiquitin-conjugating enzyme (E2), and ubiquitin ligase enzyme (E3), leading to the conjugation of the ubiquitin to a lysine (K) residue of the target protein and to the lysine residue of pre-conjugated ubiquitin (Varshavsky 1997). Therefore, the substrate protein could be attached by a single ubiquitin molecule at one lysine residue (referred as mono-ubiquitination), at multiple lysines (multi-ubiquitination), or conjugated with ubiquitin chains (poly-ubiquitination). While the K48-linked poly-ubiquitin chain usually targets proteins to proteasomal degradation, alternative linkages (e.g., K-63) are often associated with intracellular signal transduction (Glickman and Ciechanover 2002). Interestingly, the ubiquitination status of cellular proteins is also determined by a large family of proteases that remove ubiquitin and edit polyubiquitin chain (Nijman et al., 2005). These deubiquitinating enzymes (DUBs) thus affect the degradation and function of many substrates, regulating most, if not all cellular processes (Hoeller and Dikic 2009; Hussain et al., 2009).

Based on their catalytic characteristic and structural similarity, DUBs are divided into a number of classes, among which, the largest group is the ubiquitin-specific proteases (USPs) family that includes 54 members. USPs contain the characteristic catalytic domains and various ubiquitin-interacting domains and zinc-finger domains (Komander et al., 2009). Of note, it has been shown that mutations and altered expression of USPs are often associated with human cancers, indicating that it is important to characterize and understand each USP’s criticality indifferent cancers (Everett et al., 1997; Henry et al., 2003; Kennedy and D'Andrea 2006; Oliveira et al., 2004). USP47 is a 1375 amino-acid cysteine protease that contains the characteristic His and Cys catalytic domains for USPs and four ubiquitin-like domains (Piao et al., 2015). A number of studies have shown that it deubiquitinated and stabilized various substrates, including MAPK, DNA polymerase β, E-cadherin, β-catenin, SNAIL, YAP, SATB1 and β-TrCP (Parsons et al., 2011; Sako-Kubota et al., 2014; Ashton-Beaucage et al., 2016; Choi et al., 2017; Bufalieri et al., 2019; Yu et al., 2019; Pan et al., 2020). Consequently, it participates in the regulation of DNA damage repair, inflammasome activation, epithelial-mesenchymal transition, and affects proliferation and apoptosis of cancer cells, including these colorectal carcinomas (CRC).

CRC is one of the most common malignancies in both man and woman worldwide and a leading cause of cancer death (Markowitz and Bertagnolli 2009). It has been found that multiple USPs, including USP1, USP4, USP5, USP11 and USP21, were significantly increased in CRCs. They promote tumor cell growth and survival through regulating DNA damage response (Xu et al., 2019b), WNT/β-catenin pathway (Yun et al., 2015), and specific substrates such as Fos-related-antigen-1 (Yun et al., 2020), Tu translation elongation factor (Xu et al., 2019a), and protein phosphatase one catalytic subunit α (Sun et al., 2019). We found in the present study that USP47 was markedly decreased in CRCs and its reduced expression was associated with poor prognosis of CRC patients. We further identified that USP47 interacted with transcription elongation factor a3 (TCEA3, also known as TFIIS. h), and promoted its deubiquitination and stabilization. While USP47 knockdown sensitized cancer cells to anti-cancer drugs, its enforced expression increased cellular TCEA3 and chemoresistance, which was markedly attenuated by TCEA3 knockdown. Thus, USP47 functions as an effective modulator of cell death in CRC.

## Materials and Methods

### Cell Culture, Tissues and Reagents

The HEK293T cell line and human CRC cell line HCT116 were cultured in DMEM/High Glucose medium (Hyclone, Shanghai, China) supplemented with 10% of fetal bovine serum (Gibco, Shanghai, China), 100 U/ml of penicillin, 100 μg/ml of streptomycin at 37°C in a humidified incubator containing 5% CO_2_, 95% air atmosphere. The GSDME-knockout HCT116 (GSDME-KO) cells were generated by using the CRISPR-Cas9 system with lenti-CRISPR-v2 vector as previously described (PMID: 24157548). The sequence of the guide RNA was as follow: 5′-AAG​TTT​GCA​AAC​CAC​GTG​AG-3′. The 21 pairs of CRC and para-cancerous normal tissues were collected with informed consensus from patients enrolled into the First Affiliated Hospital of Soochow University, Suzhou, China. The study was approved by the Ethical Review Board of the First Affiliated Hospital of Soochow University. The tissue array was made from 136 CRC tissues collected with informed consensus from surgical specimens of patients enrolled into in the Department of Colorectal and Anal Surgery, Xinhua Hospital, Shanghai Jiaotong University School of Medicine, from January 2008 to December 2016. The collection was approved by Xinhua Hospital Ethics Committee. The clinicopathological information of these patients were shown in Table 1. Doxorubicin, MG132 and P22077 were purchased from Selleck Chemicals (Shanghai, China).

**TABLE 1:** Case information.

Clinical parameters		Case (%)
Gender	Male	69 (50.7)
	Female	67 (49.3)
Age (year)	≤65	66 (48.5)
	>65	70 (51.5)
Stage	I	15 (11.0)
	II	95 (69.9)
	III	26 (19.1)
T	1	2 (1.5)
	2	15 (11.0)
	3	46 (33.8)
	4	73 (53.7)
N	0	68 (50.0)
	1	41 (30.1)
	2	27 (19.9)
M	0	125 (91.9)
	1	11 (8.1)
USP47	High	77 (56.6)
	Low	59 (43.4)

### Plasmid Construction and siRNA Sequences

The pLVX-Flag-USP47 plasmid was purchased from GeneCopoeia (Guangzhou, China). TCEA3 cDNA (GenBank accession number: NM_003196.3) was amplified and cloned into pcDNA3.1. The sequences of siRNAs targeting USP47 were as follows: siRNA#1 sense: 5-GCU​GUC​GCC​UUG​UUA​AAU​ATT-3, antisense: 5- UAU​UUA​ACA​AGG​CGA​CAG​CTT-3; siRNA#2 sense: 5-GGC​GUC​AAG​UCA​ACA​UAU​ATT-3, antisense: UAU​AUG​UUG​ACU​UGA​CGC​CTT-3; siRNA#3 sense: 5-CCA​GCA​AUC​AAG​AGU​UUG​ATT, antisense: 5-UCA​AAC​UCU​UGA​UUG​CUG​GTT-3. The sequences of siRNAs targeting TCEA3 were as follows: siRNA#2 sense: 5-GGG​ACA​AGU​GUG​UGG​AGA​UTT-3, antisense: 5- AUC​UCC​ACA​CAC​UUG​UCC​CTT-3; siRNA#3 sense: 5-CCU​CUU​CCA​GUG​CAG​CAA​ATT-3, antisense: 5-UUU​GCU​GCA​CUG​GAA​GAG​GTT-3.

### Immunoblotting and Antibodies

The cultured cells and the human tissues were lysed with the appropriate amount of RIPA lysis buffer (Beyotime, Shanghai, China) containing 1 ×protease inhibitor mixture (Roche) and PMSF (Beyotime, Shanghai, China). The proteins in the supernatants were subjected to SDS-PAGE and immunoblotting as described previously (Hou et al., 2019). The antibodies used were as follows: anti-PARP (Cell Signaling Technology), GSDME (Abcam), USP47 (Santa Cruz), GAPDH (Abgent Biotechnology), β-actin (Santa Cruz), α-Tubulin (Santa Cruz), Flag (Medical and Biological Laboratories), Myc (Medical and Biological Laboratories), Ub (Santa Cruz), Bax (Santa Cruz).

### RNA Extraction and RT-qPCR

Total RNA was extracted using the TRIzol reagent (X. Hou et al., 2019). First Strand cDNA Synthesis Kit and Fast-Start universal SYBR Green master mix were purchased from TakaraBio (Dalian, China). The following primers were used for real-time PCR: USP47, sense: 5-CAG​TGG​GAT​TCC​TTT​GGA​TG -3, antisense: 5-GGC​CAG​ACA​TTC​AGG​GTA​GA -3; and TCEA3, sense: 5- TCG​CTG​GAA​GTT​CTG​CTG​ATG​G -3, antisense: 5- ATT​CTC​CAA​TTA​GGC​TCC​CCC​A -3; β-actin, sense: 5- GCG​GGA​AAT​CGT​GCG​TGA​CAT​T -3, antisense: 5- GAT​GGA​GTT​GAA​GGT​AGT​TTC​G -3; Bax, sense: 5- CAT​GGG​CTG​GAC​ATT​GGA​CT -3, antisense: 5- AAA​GTA​GGA​GAG​GAG​GCC​GT -3. The mRNA levels of the target genes were normalized to β-actin. Data were analyzed using GraphPad Prism 5.

### Immunoprecipitation (IP) and Silver Staining

The cultured cells were lysed with the appropriate amounts of IP lysis buffer (50 mm HEPES at pH 7.5, 150 mm NaCl, 1.5 mm MgCl_2_, 10 mm NaF, 1 mm EGTA, 1% Triton X-100, 10% Glycerol) containing 1× protease inhibitor mixture (Roche) and PMSF (Beyotime, Shanghai, China). The lysates were incubated with appropriate amount of primary antibody overnight at 4°C and then mixed with protein A/G-Sepharose beads (Santa Cruz) for 2 h. After 5 times of washing, the beads were boiled in 2 × SDS loading buffer for 10 min and subjected to SDS-PAGE. Following careful washing with clean water, silver staining of the gel was performed according to the manufacturer’s instructions (Roche). The visible differential protein bands were excised and prepared for analyzing with LC-MS/MS, which was conducted by ProtTech (Wuhan, China).

### Immunohistochemistry

The immunostaing of the CRC tissue array was performed with a anti-USP47 Ab (Santa Cruz). The staining intensity was graded as follows: 0, negative; 1, weak; 2, moderate; and 3, strong; and the percentage of positively stained cells was recorded as follows: 0, <5%; 1, 5–25%; 2, 26–50%; 3, 51–75%; and 4, >75% (Wei et al., 2018). Five fields were randomly selected in each section. The score was calculated by multiplying the staining intensity number with the percentage of positively stained cells’ number. Total immunohistochemical scores of 0–six were considered to be low, whereas scores of 7–12 were considered to be high.

### Statistical Analysis

Statistical analyses were performed using the GraphPad Prism. Data were presented as the means ± standard deviation (SD). The paired, two-tailed Student’s t-test or one-way ANOVA were used to assess the significance between two groups. All reported differences were ^*^ *p* < 0.05, ^**^ *p* < 0.01, ^***^ *p* < 0.001 unless otherwise stated.

## Results

### USP47 Expression Is Reduced in Primary CRC and Associated With Disease-Free Survival of CRC Patients

**FIGURE 1:**
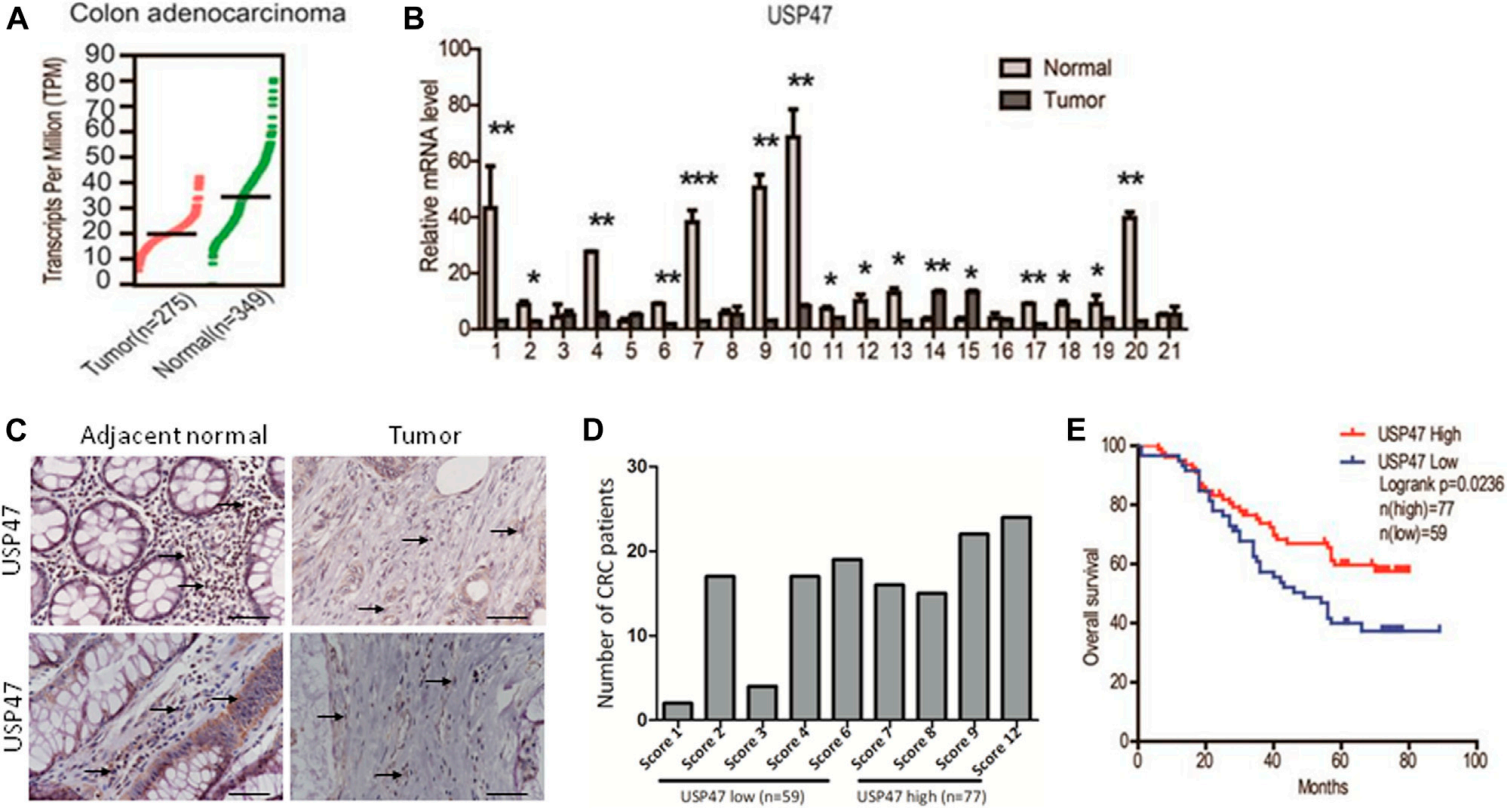
Decreased expression of USP47 is associated with shorter disease-free survival time of CRC patients. **(A)** USP47 expression levels in colon adenocarcinoma and adjacent normal tissues retrieved from GEPIA database. Log2 (TPM+1) transformed data was used in the plot. **(B)** The mRNA levels of USP47 in 21 pairs of CRC and adjacent normal tissues were quantitated by RT-qPCR. The relative levels of the target genes were normalized to that of β-actin. The data were independently repeated three times and presented as the means ± SD, ^*^ *p* < 0.05, **p < 0.01. **(C)** Anti-USP47 immunohistochemistry of CRC and adjacent normal tissues. **(D, E)** Analyses of anti-USP47 immunostaining scores **(D)** and the disease-free survival of CRC patients **(E)**. CRC, colorectal cancer.

We retrieved the data of USP47 expression measured by RNAseq from the public database: Gene expression profiling interactive analysis (GEPIA). As shown in Figure 1A, the TPM (Transcripts per kilobase of exon model per million mapped reads) of USP47 was significantly reduced in colon adenocarcinoma (n = 275) than the normal tissues (n = 349). We also collected 21 pairs of surgical excised tumor and adjacent normal tissues from patients with CRC. The levels of USP47 mRNA were quantified by using RT-qPCR with GAPDH as a control. Comparing that of non-cancerous tissues, the relative mRNA level of USP47 was markedly decreased in 14 cases of CRC tissues (66.7%). Only two cases showed increased expression of USP47 (9.5%), 5 cases had no significant changes in CRC (23.8%) (Figure 1B). The decreased level of USP47 in colorectal cancer was also evident when examined by immunohistochemistry (Figure 1C). Analyses of a tissue array containing 136 CRC specimens showed that 77 specimens had a high score (7–12), and 59 had a low score (0–6) (Figure 1D; Table 1). Patients with low expression of USP47 had a shorter disease-free survival compared with those with higher level (Figure 1E). In addition, USP47 expression was significantly associated with disease stages, but not patients’ gender and age (Table 2). Thus, while increased expression of USP1, USP4, USP5, USP11, and USP21 promote CRC development (Xu et al., 2019b), decreased USP47 is associated with the development of CRC.

**TABLE 2:** Correlation between USP47 levels in CRC patients and their clinicopathologic characteristics.

Tissues	Cases(N)	USP47 expression		*p*
		Low	High	
**Gender**				
Male	69	29	40	0.6537
Female	67	30	37	
**Age (year)**				
≤65	66	28	38	0.7616
>65	70	31	39	
**Stage**				
I + II	110	50	60	0.0376*
III	26	9	17	

*p < 0.05 was considered statistically significant.

### USP47 Regulates CRC Cell Death Induced by Doxorubicin

**FIGURE 2:**
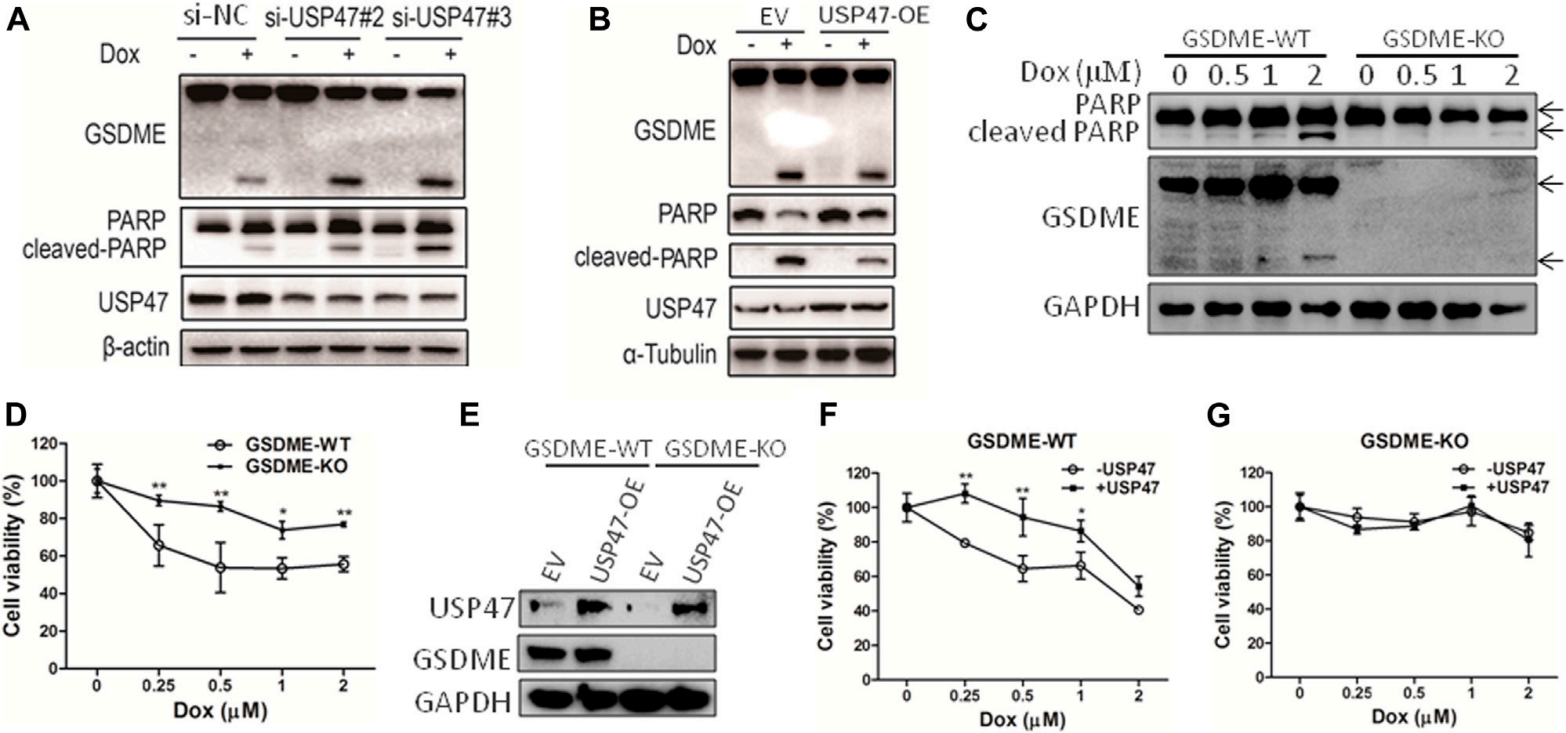
USP47 regulates the cell death of CRC cells induced by doxorubicin. **(A)** Immunoblotting of GSDME, PARP and USP47 in HCT116 cells transfected with siRNAs for USP47 and treated with 1 μM Dox, doxorubicin. **(B)** Immunoblotting of GSDME, PARP and USP47 in HCT116 cells overexpressing USP47 and treated with 1 μM. **(C, D)** The wild-type (GSDME-WT) and GSDME-knockout (GSDME-KO) HCT116 cells were incubated with indicated Dox overnight, and cells were prepared for immunoblotting **(C)** and CCK-8 assay **(D)**. ^*^ *p* < 0.05, **p < 0.01. **(E)** The overexpressing efficacy of USP47. **(F, G)** GSDME-WT **(F)** or GSDME-KO **(G)** HCT116 cells were transfected with empty vector or USP47-overexpressing plasmids for 24 h, and then transfected cells were incubated with indicated Dox overnight, followed by CCK-8 assay. ^*^ *p* < 0.05, **p < 0.01.

As found in primary CRCs, some colorectal cancer cell lines expressed detectable USP47. To assess the function of USP47, HCT116 cells were transfected with siRNAs targeting USP47 in the absence or presence of anti-cancer drug doxorubicin (Dox). While knockdown of USP47 alone did not have notable effects on HCT116 in 24 h, Dox-induced cell death was markedly enhanced (Supplementary Figure S1A). Of note, the number of “bubble-like” dead cells in Dox-treated culture was significant increased upon USP47 knockdown (Supplementary Figure S1B). As the appearance of “bubble-like” may be associated with pyroptosis, we examined the activation of gasdermin (GSDME), a marker of pyroptosis, in the HCT116 cells exposed to various treatments. As shown in Figure 2A, GSDME cleavages were markedly increased in cells treated with Dox and USP47-trageting siRNAs. Interestingly, the cleavages of PARP, a marker of apoptosis, were also significantly enhanced, suggesting that these cells died by both pyroptosis and apoptosis (Figure 2A). We then transfected HCT116 cells with USP47-expressing vector and examined their responses to the stimuli. As shown in Figure 2B, overexpression of USP47 decreased effectively the changes of the markers for pyroptosis and apoptosis. To further assess the role of GSDME, GSDME-KO cells were generated, and these cells were more resistant to Dox, manifested as reduced PARP cleavages and cell viability reduction (Figures 2C,D). Moreover, enforced expression of USP47 did not significantly reduce the cytotoxicity of Dox in GSDME-KO cells (Figures 2E–G). Above results indicated that USP47 mediated the pyroptosis and apoptosis induced by Dox in CRC.

### USP47 Regulates the Expression of TCEA3

**FIGURE 3:**
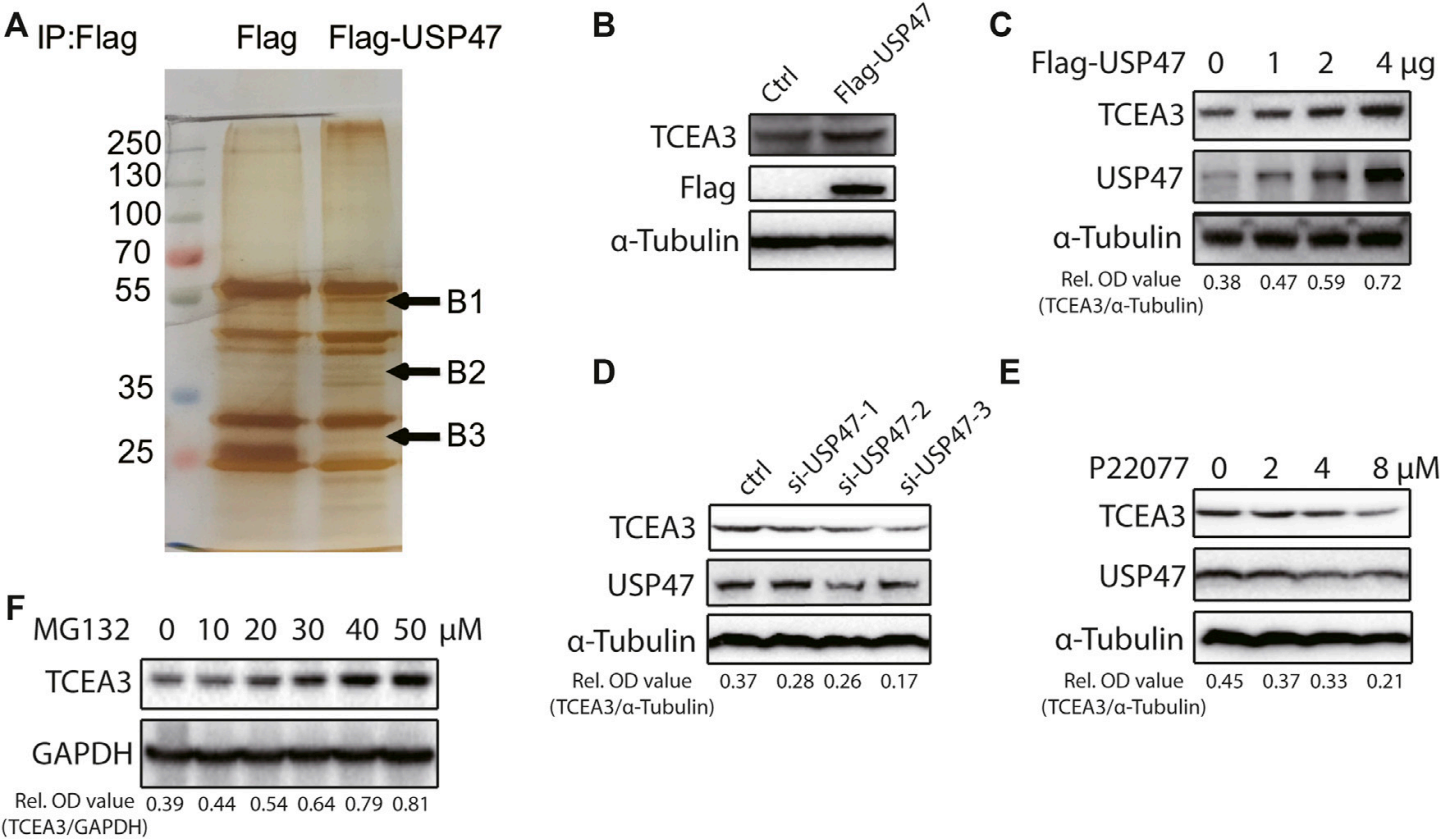
USP47 regulated the expression of TCEA3. **(A)** Silver staining of lysates from HEK293T cells transfected with Flag-USP47-expressing vector for 48 h. **(B)** Anti-Flag immunoblotting of HCT116 cells transfected with Flag-USP47-expressing vector for 48 h. **(C)** The expression of USP47 and TCEA3 in HCT116 cells transfected with indicated amounts of USP47-expressing plasmid. **(D)** The expression of TCEA3 in USP47 HCT116 cells transfected with siRNAs against USP47. **(E)** Immunoblotting of TCEA3 in HCT116 cells treated with indicated amounts of USP47 inhibitor P22077 for 24 h. **(F)** Immunoblotting of TCEA3 in HCT116 cells treated with indicated amounts of MG132 for 6 h.

The effects of USP47 on CRC cell death propelled us to explore the underlying mechanisms. We transfected 293T cells with vector expressing Flag or Flag-USP47 and immunoprecipitated with an anti-Flag antibody to find potential substrate proteins. The immunoprecipitates were separated by SDS-PAGE and subjected to silver staining. Three visible differential bands were then excised and prepared for GC-MS (Figure 3A). Interestingly, TCEA3 (transcription elongation factor a3) was identified as one of the proteins in band 2. When HCT116 cells were transfected with indicated amounts of USP47-expressing vectors, the level of TCEA3 was increased markedly and dose-dependently (Figures 3B,C). On the other hand, both USP47 knockdown and USP47 inhibitor P22077 reduced the expression of TCEA3 (Figures 3D,E). Furthermore, proteasome inhibitor MG132 treatment increased the level of TCEA3 dose-dependently (Figure 3F). These results indicated that TCEA3 undergoes proteasomal degradation in CRC cells, which could be regulated by USP47.

### USP47 Deubiquitinates and Stabilizes TCEA3

**FIGURE 4:**
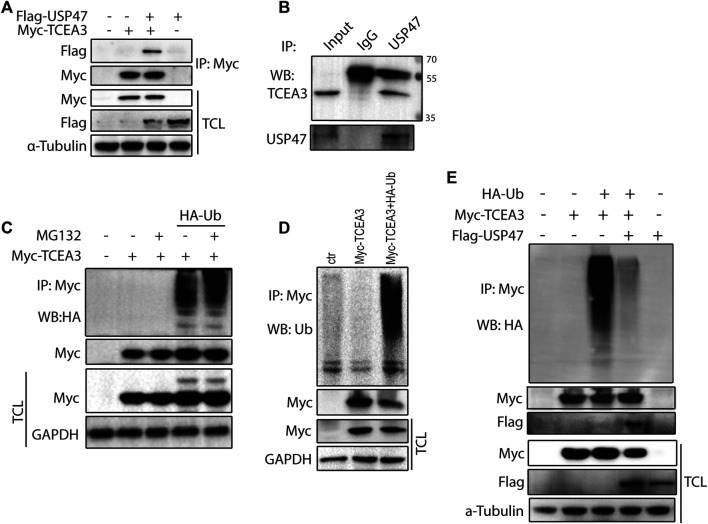
USP47 acts as a deubiquitinating enzyme of TCEA3. **(A)** co-immunoprecipitation of overexpressed USP47 and TCEA3. Flag-USP47 and Myc-TCEA3-expressing plasmids were transfected into HEK293T cells for 48 h. Total cell lysate was immunoprecipitated with anti-Myc antibody and blot with anti-Flag antibody. Total cell lysates were also directly blotted with the antibodies. **(B)** The interaction of endogenous USP47 and TCEA3. Lysates from HCT116 cells were immunoprecipitated with anti-USP47 antibody and blotted with anti-TCEA3 antibody **(C, D)** The ubiquitination of TCEA3. Myc-TCEA3 and HA-Ub plasmids were transfected into HEK293T cells for 48 h. After exposed to 20 μM of MG132 for 6 h, the cells were harvested, immunoprecipitated with anti-Myc antibody, and blotted with anti-HA **(C)** or anti-Ub antibodies **(D)**. **(E)** USP47 decreased the ubiquitiantion of TCEA3. Flag-USP47, Myc-TCEA3 and HA-Ub plasmids were transfected into HEK293T cells for 48 h. Following treatment with 20 μM of MG132 for 6 h, cell lysates were immunoprecipitated with anti-Myc antibody and blotted with anti-HA, anti-Myc or anti-Flag antibody.

To further understand how USP47 regulated TCEA3, we co-tranfected vectors expressing Myc-TCEA3 and Flag-USP47 into HEK293T cells and performed immunoprecipitation with the anti-Myc antibody. As shown in Figure 4A, Flag-tag protein USP47 was effectively pulled down by the anti-Myc antibody when Myc-tagged protein TCEA3 was co-expressed. Furthermore, an anti-USP47 antibody was able to immunoprecipitated endogenous USP47 as well as TCEA3 in HCT116 (Figure 4B). These data demonstrated that USP47 and TCEA3 interacted in CRC cells. As USP47 is a DUB, we transfected cells with vector expressing HA-Ub and Myc-TCEA3 for 24 h and treated the cells with proteasome inhibitor MG132 for 6 h. Following IP with the anti-Myc antibody, poly-ubiquitination of TCEA3 was detected with both anti-HA (Figure 4C) and anti-Ub antibodies (Figure 4D), especially in cells exposed to MG132, indicating that TCEA3 undergoes ubiquitination and proteasomal degradation in HCT116 cells. Interestingly, co-transfection of USP47 with TCEA3 significantly reduced the ubiquitiantion of TCEA3 (Figure 4E), demonstrating that USP47 was capable of deubiquitinating TCEA3.

### USP47 Regulates the Pyroptosis and Apoptosis Through the Substrate TCEA3

**FIGURE 5:**
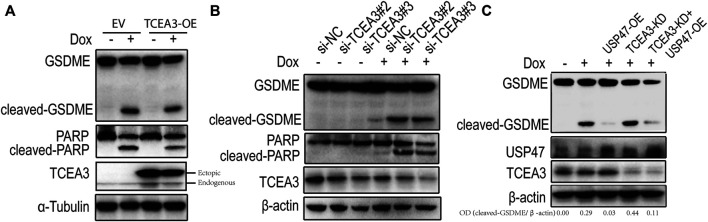
TCEA3 mediated USP47-induced enhancement of pyroptosis and apoptosis. **(A)** GSDME, PARP and TCEA3 in HCT116 cells over-expressing TCEA3. After transfected with TCEA3-expressing plasmid for 1 day, the HCT116 cells were treated with 1 μM of Dox for 24 h and examined by immunoblotting. **(B)** GSDME, PARP and TCEA3 in HCT116 cells with TCEA3 knockdown. **(C)** Examination of GSDME, USP47 and TCEA3 in HCT116 cells treansfected with siRNA targeting TCEA3 and plasmid expressing USP47. TCEA3-OE, TCEA3-overexpressing plasmid; USP47-OE, USP47-overexpressing plasmid; TCEA3-KD, siRNA targeting TCEA3 (si-TCEA3#3).

To assess the potential role of TCEA3 in USP47-meidated pyroptosis and apoptosis of CRC, we transfected vector expressing TCEA3 or siRNA targeting TCEA3 into CRC cells. While overexpression of TCEA3 attenuated the cleavages of GSDME and PARP induced by Dox (Figure 5A), TCEA3 knockdown increased the cleavages of GSDME and PARP (Figure 5B). We then knocked down TCEA3 in USP47-overexpressing CRC cells. As shown in Figure 5C, USP47 overexpression reduced Dox-induced GSDME cleavages (Lane 3), which were mitigated by TCEA3 knockdown (Lane 5). These data indicated that TCEA3 was an important mediator for the action of USP47 on pyroptosis and apoptosis induced by Dox.

### Bax Is Involved in the Pyroptosis and Apoptosis Regulated by TCEA3

**FIGURE 6:**
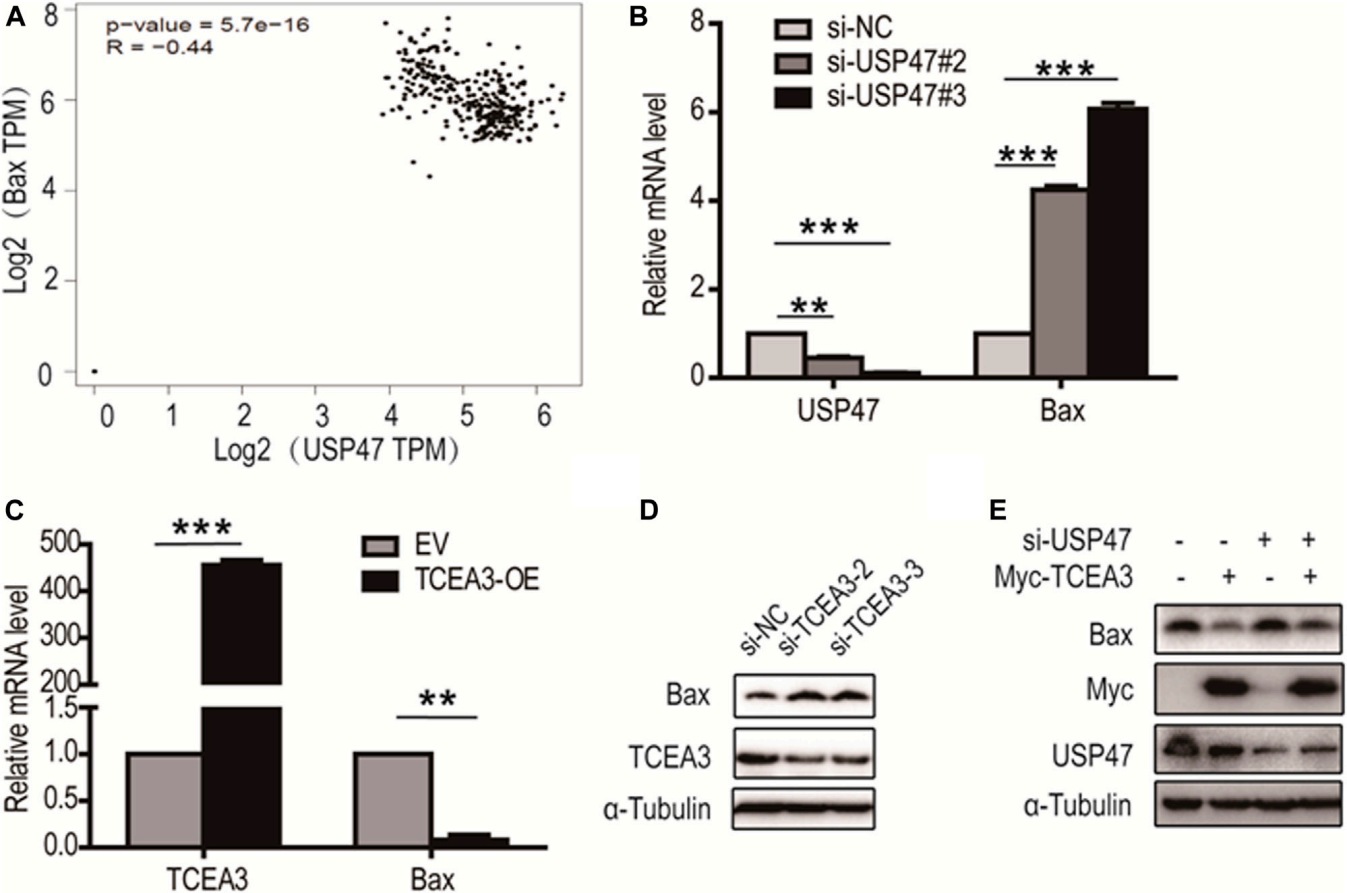
Regulation of Bax transcription by TCEA3. **(A)** USP47 and Bax expressions were correlated. Data were retrieved from GEPIA database. **(B)** Bax mRNA level in HCT116 cells was increased after the transfection of USP47-targeted siRNAs for 24 h **p < 0.01, ***p < 0.001. **(C)** The mRNA level of Bax was reduced in HCT116 cells after the transfected of TCEA3-expressing plasmid for 24 h **p < 0.01, ***p < 0.001. **(D)** Bax was increased in HCT116 cells following transfection with TCEA3-targeted siRNAs for 48 h as examined by anti-Bax immunoblotting. **(E)** The expression of Bax in HCT116 cells transfected with TCEA3-expressing plasmid and/or USP47-targeted siRNAs for 48 h.

From analyzing USP47-associating genes involved in pyroptosis and apoptosis in GEPIA database, it was found that Bax, a predominant pro-apoptotic molecule, was one of the proteins negatively correlated with USP47 expression (Figure 6A). We therefore examined whether USP47 affected the expression of Bax in CRC cells. As shown in Figure 6B, knockdown of USP47 resulted in a marked up raise of Bax mRNA as determined by RT-qPCR. Whereas enforced expression of TCEA3 led to a significant reduction of Bax mRNA (Figure 6C). Immunoblotting analyses also revealed that TCEA3 knockdown increased cellular Bax (Figure 6D), and TCEA3 overexpression decreased Bax at protein level (Figure 6E). Furthermore, when USP47 was silenced, the inhibitory of TCEA3 overexpression on Bax expression was mitigated (Figure 6E, Lane 4). These results demonstrated that the expression of Bax was regulated by USP47 through TCEA3. Thus, USP47-TCEA3-Bax axis was an effective regulator for the pyroptosis and apoptosis of CRC cells, and may be served as a novel target for effective chemotherapy.

## Discussion

Transcription of many human genes is dependent on the action of RNA polymerase II, a multiple subunits complex. TCEA3 is one of three transcription elongation factors in vertebrates, which promotes the mRNA cleavage by enhancing the intrinsic nuclease activity of RNA polymerase II (Kettenberger et al., 2003). TCEA3 has been found to be highly expressed in embryonic stem cells to regulate the differentiation of the cells (Park et al., 2013). More recent studies showed that it is also expressed in many tissues, including muscle, adrenal prostrate, ovary, colon, and liver. It has been also found that TCEA3 was downregulated in a variety of cancers types, and the lower TCEA3 expression is associated shorter overall survival of cancer patients (Kazim et al., 2020). Our finding in this study that USP47 is able to deubiquitinate and stabilize TCEA3 indicates that the reduced expression of USP47 may play a significant role in the reduction of TCEA3 in cancers, and it is of great interesting to further identify the E2 and E3 that ubiquitinate TCEA3 in the future. Interestingly, it has been reported that the mRNA level of USP47 was increased in a small group of colon mucosae from early onset CRC patients (Pan et al., 2020). USP47 was also found deubiquitinating YAP in the same study, suggesting that the deubiquitinating enzyme may also affect cell growth and tumor initiation under certain circumstances.

The relationship between TCEA3 and cell death appears complicated. Knockout of TCEA in mice is embryonic lethal, likely due to the dramatic increase of apoptotic cells, which was found in the fetal liver and other tissues (Ito et al., 2006). It has also been shown that knockdown of TCEA3 inhibited the proliferation of breast cancer cells and induces apoptosis (Hubbard et al., 2008), indicating that the protein is required for the survival of cells. However, it has also been found that enforced expression of TCEA3 inhibited proliferation and induced apoptosis in a number lines of cancer cells, including RMS, HeLa, PC3, MCF7, and MDA-321 (Kazim et al., 2020). In these cells, TCEA3 activated both intrinsic and extrinsic pathways of apoptosis. Of note, they all derived from tissues expressing high level TCEA3, suggesting that TCEA3 is likely suppressed during tumor development to block apoptosis. In our study of colon cancer cells, TECA3 knockdown or overexpression alone did not affect cell death markedly. When the cells were exposed to cytotoxic agent Dox, TCEA3 as well as USP47 protect them from apoptosis and pyrotosis, whereas knockdown of TCEA3 enhances Dox-induced cell death. Taking together, these results indicate that the effects of TCEA3 on cell death is highly cell type-dependent. Given the protein as a transcription elongation factor, it is conceivable that its influence on cells depends on driving mutations in cancer cells, status of the cell death machinery, and the damage of drug on the cells.

Pyroptosis is a form of programmed cell death characterized by cleavage of gasdermin family members, cell swelling, and formation of bubble-like protrusions (Shi et al., 2017). While pyroptosis holds back tumor development, it also provokes inflammation, which often provides a favorable environment for tumor initiation and progression. Therefore, a variety of efforts have been made to find agents that induce pyroptosis as anti-cancer therapeutics, which might overcome the resistance to apoptosis that occurred in many cancer cells. It has also become evident that effective anti-tumor chemotherapies often depend on their toxic action on tumor cells as well as host immune response (Hou et al., 2020). Recent studies further demonstrated that pyroptosis of tumor cells and the release of HMGB1 are able to induce effective antitumor immunity (Wang et al., 2020). Moreover, induction of pyroptosis in tumor cells sensitized tumors to immune checkpoint blockade. In this study, we found that the apoptosis and pyroptosis were both induced in CRC cells by Dox, and knockdown of USP47 or TCEA3 enhanced markedly pyroptotic cell death. Intriguingly, USP47 knockdown enhanced Dox-induced GSDME cleavages, which has been shown to be mediated by caspase 3, but we did not found markedly increased activated caspase three in the system. It was conceivable that the substrates of USP47 were able to protect GSDME from cleavages by caspase 3. The activity of caspase-3 may be increased by knockdown of USP47 or TCEA3 indirectly. Thus, this is an interesting system to explore the mechanisms of pyroptosis induction in tumor cells in our future work. Further, it is conceivable that inhibitors of USP47 and TCEA3 might be effective agents to enhance various chemotherapeutics-induced pyroptosis and increase the effectiveness of anti-tumor therapies. These results indicated that the USP47-TCEA3 axis may modulate cell pyroptosis and apoptosis and could be served as a target for therapeutic intervention in CRC.

## Data Availability

The original contributions presented in the study are included in the article/Supplementary Material, further inquiries can be directed to the corresponding authors.
