# Supplementary material for: USP47-Mediated Deubiquitination and Stabilization of TCEA3 Attenuates Pyroptosis and Apoptosis of Colorectal Cancer Cells Induced by Chemotherapeutic Doxorubicin
Source: Front Pharmacol. 2021 Sep 23;12:713322. doi: 10.3389/fphar.2021.713322 (PMC8495243; doi:10.3389/fphar.2021.713322)
Supplement: Supplementary file 1 [file DataSheet1.docx]

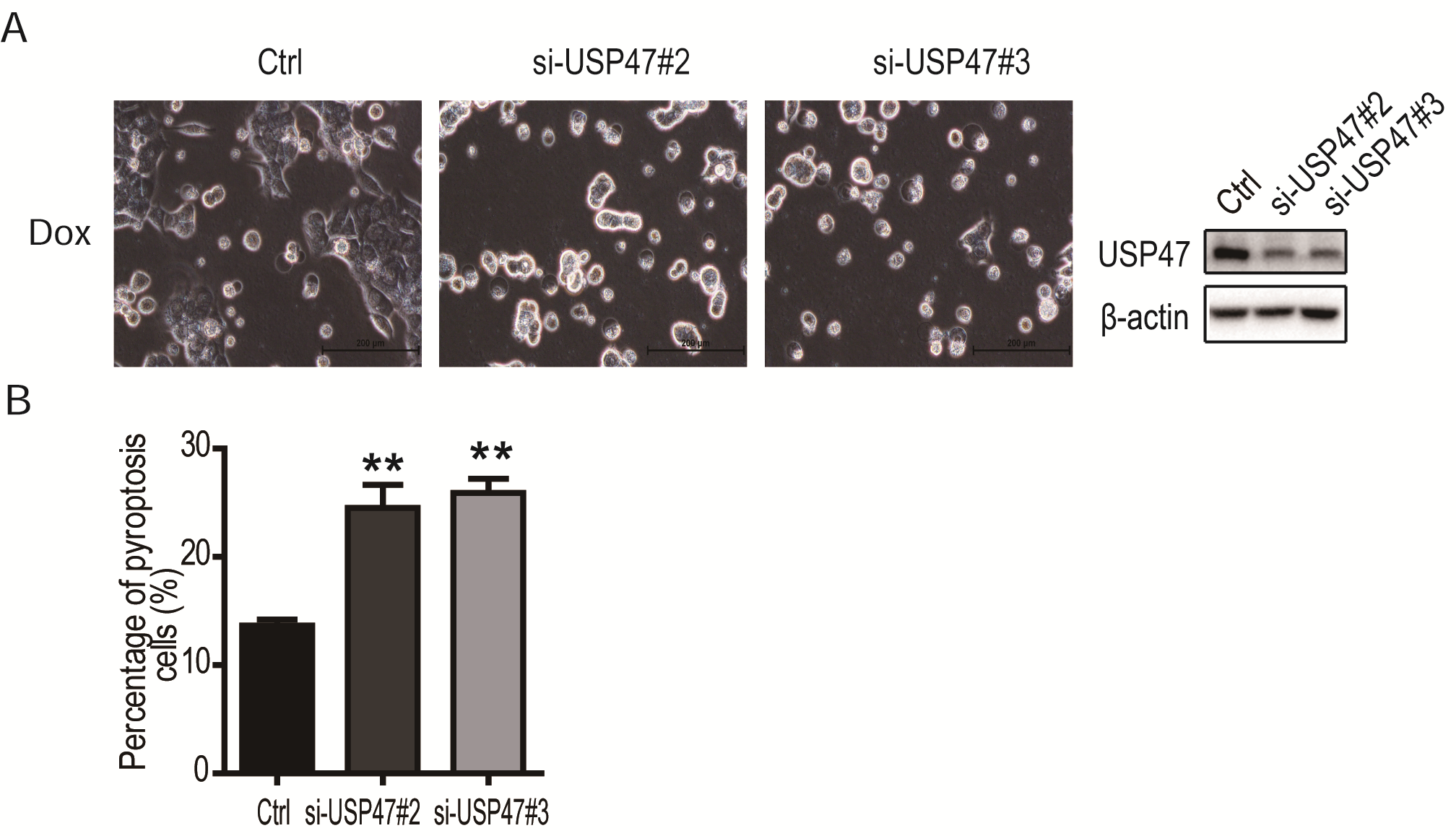


**Supplementary Figure 1. Silencing of USP47 increased the pyroptosis by Doxorubicin in CRC cells. (A)** USP47 in HCT116 cells was knockdown effectively with our siRNA#2 and #3. After transfected with the siRNA for 1 day, HCT116 cells were treated with 1 μΜ of doxorubicin for 24 hours. Photos taken under microscope indicated the presence of pyroptotic cells, especially in culture treated with. USP47 –targeting siRNA and doxorubicin. **(B)** The percentages of pyroptotic cells in USP47 knockdown HCT116 treated with doxorubicin. After siRNA transfection for 1 day, HCT116 cells were treated with 1 μΜ of doxorubicin for 24 hours. The “bubble-like” cells were counted as pyroptotic cells in 5 fields under microscope and normalized to the total cells in the fields.
